# Secure attachment priming inhibits the generalization of conditioned fear

**DOI:** 10.1186/s40359-024-01857-9

**Published:** 2024-06-19

**Authors:** Xu Li, Yong Yang, Ranran Wang, Lehong Zhou, Xifu Zheng

**Affiliations:** 1 Center for Counseling and Psychological Services, Guangdong University of Petrochemical Technology, Maoming, Guangdong 525000 China; 2 School of Educational Science, Xinyang Normal University, Xinyang, China; 3 School of Psychology, South China Normal University, Guangzhou, China

**Keywords:** Fear generalization, Secure attachment priming, Skin conductance response, Inhibitory effect

## Abstract

**Background:**

Fear overgeneralization constitutes a susceptibility factor contributing to the development and maintenance of anxiety spectrum disorders. Extant research has demonstrated that exposure to positive and supportive social relationships attenuates fear acquisition and promotes the extinction of conditioned fear responses. However, the literature lacks investigation into the effect of secure attachment priming on inhibiting the generalization of conditioned fear.

**Methods:**

In this study, college students were recruited via online platforms to voluntarily engage in the experimental procedures, resulting in 57 subjects whose data were deemed suitable for analysis. The experimental protocol consisted of four consecutive phases: pre-acquisition, acquisition, priming, and generalization. The priming phase consisted of two experimental conditions: secure attachment priming (experimental group) and positive emotion priming (control group). This study adopted the perceptual discrimination fear conditioning paradigm, employing subjective expectancy of shock ratings and skin conductance responses as primary assessment indices. Individual difference variables were measured using corresponding psychological measurement scales.

**Results:**

In terms of generalization degree, a notable divergence surfaced in the skin conductance responses across various generalization materials between the secure attachment priming group and the control group. Similarly, during generalization extinction, a significant disparity emerged in the skin conductance responses across different generalization phases between the secure attachment priming group and the control group. In addition, individual differences analyses revealed that the inhibitory effect of secure attachment priming on fear generalization was not affected by intolerance of uncertainty and attachment orientations. Conversely, slope analyses confirmed that as intolerance of uncertainty increased, the inhibitory effect of positive emotion priming on fear generalization was attenuated.

**Conclusion:**

The findings suggest that activating participants' representations of secure attachment via imagination effectively attenuates the generalization of perceptual fear at the physiological level. The inhibitory effect of secure attachment priming appears to be distinct from positive emotional modulation and remains unaffected by individual trait attachment styles. These results offer novel insights and avenues for the prevention and clinical intervention of anxiety spectrum disorders.

**Supplementary Information:**

The online version contains supplementary material available at 10.1186/s40359-024-01857-9.

## Introduction

Fear learning facilitates individuals in promptly identifying environmental threats and initiating defensive responses, which are imperative for human adaptation to intricate survival circumstances [1]. However, Human fear learning is not limited to situations or things directly related to fear, any stimuli closely resembling the original threat can elicit fear responses, leading to fear generalization [2]. Moderate fear generalization heightens individual vigilance, evading potential threats and swiftly anticipating and managing hazardous environments [3]. Nevertheless, excessive generalization to neutral cues unrelated to the original threatening stimulus depletes individuals' attentional resources, potentially contributing to anxiety spectrum disorders such as generalized anxiety disorder (GAD), post-traumatic stress disorder (PTSD), panic disorder (PD), specific phobia, among others [4].

Given that the excessive generalization of fear is a potential cause of anxiety spectrum disorders, it is therefore crucial to explore methods and clinical intervention techniques to inhibit the excessive generalization of fear in humans. Humans are social animals, and seeking support from others in social interactions to buffer the fear induced by threatening stimuli is a fundamental survival strategy for humans. Previous studies have found that presenting positive and supportive social relationships, such as social support images [5] and attachment sounds [6], can promote the inhibition of fear emotions. Even observing the safe behavior of others can inhibit the recovery of conditioned fear [7]. In terms of inhibiting fear generalization, previous studies have also found that the presence of others can inhibit fear generalization [8], and oxytocin produced in intimate relationships has been found to inhibit the generalization of conditioned fear [9].

Attachment refers to the profound emotional bond established between individuals and their primary caregivers during development, serving as the foundation for forming and developing their social relationships. Operating as an adaptive behavioral system, attachment functions as a "Safe Haven" and a "Secure Base", significantly contributing to individuals' sense of security [10]. Individuals with diverse attachment styles harbor distinct internal representations of self, others, and relationships, manifesting variations in everyday life's social behaviors, emotional expressions, and cognitive tendencies [11]. Mikulincer and Shaver proposed and formulated an Integrative Model of Attachment-System Dynamics, elucidating the disparities in emotional regulation strategies embraced by individuals with distinct attachment styles upon activation of the attachment system [12–14]. This model encompasses secure attachment, hyperactivation, or deactivation strategies. When faced with threats, Securely attached individuals employ secure attachment strategies, seeking solace and aid from attachment figures while utilizing them as a springboard for navigating the world, enhancing their social competence. Conversely, individuals grappling with attachment anxiety and avoidance resort to hyperactivation and deactivation strategies, respectively, as mechanisms to cope with and alleviate stress [15].

With the shift of attachment research toward social cognition, scholars have further developed the Integrative Model of Attachment-System Dynamics. By introducing the priming paradigm into attachment research, researchers have dynamically examined the real-time effects of secure attachment priming on individuals with different attachment styles. This was achieved by contextually activating participants' representations of secure attachment through secure-base priming techniques. Attachment representations mainly encompass positive psychological images of attachment figures and positive interaction experiences, such as perceiving them as accessible, responsive, and sensitive. A study by Mikulincer et al. demonstrated that participants exhibited more compassionate behaviors after viewing images depicting intimate interactions between mothers and infants [16]. This indicates that exposure to real or imagined secure attachment stimuli can activate individuals' sense of attachment security, temporarily producing positive effects similar to trait attachment. Subsequent research has also found that activating secure attachment representations in different ways can effectively help individuals cope with stress and threats, enhance feelings of security, improve interpersonal relationships, and regulate emotions, thus demonstrating the “Broaden-and-Build” effect of secure attachment [17]. Research in cognitive neuroscience has found that hand-holding and the presence of a secure attachment figure (e.g., romantic partner) can also reduce fear-related activation in the brain [18, 19]. Toumbelekis et al. were the first to apply secure attachment priming to inhibit conditioned fear acquisition [20], facilitate the extinction of conditioned fear [21], and suppress the reconsolidation of fear memories [22]. They discovered positive effects independent of positive emotion priming, yet there is currently no research exploring the effects of secure attachment priming on inhibiting the generalization of conditioned fear.

Several studies have explored the influence of individual differences on conditioned fear. Toumbelekis et al. utilized attachment orientations as predictor variables and investigated their effects on fear acquisition and extinction across different priming types. Their findings revealed that individual attachment styles did not significantly impact the effects of various priming types on fear acquisition and extinction[20, 21]. However, the impact of secure attachment priming on fear memory reconsolidation diminished with increased attachment anxiety [22]. Furthermore, the role of intolerance of uncertainty in the generalization of conditioned fear has also been a subject of interest, with researchers noting that individual differences in intolerance of uncertainty play a critical role in conditioned fear generalization [23]. Morriss et al. discovered that higher intolerance of uncertainty diminishes individuals' ability to differentiate between safety and threat cues, resulting in an overestimation of the threat value of safety cues during the conditioned fear process, thereby heightening the risk of fear generalization[24]. Additionally, Bauer et al. found that higher intolerance of uncertainty improves the ability to discriminate between threat and safety cues, but is still linked to a greater degree of fear generalization [25, 26].

Based on perceptual fear generalization gradients, researchers can assess the extent of discrimination learning and fear generalization [27]. The Discrimination Fear Conditioning Paradigm stands as a cornerstone in studying perceptual fear generalization [28]. This paradigm employs a solitary sensory cue stimulus (e.g., circles of varying sizes) as both the conditioned stimulus (CS) and the generalization stimulus (GS). The CS+ is repeatedly paired with a fear-inducing stimulus (unconditioned stimulus, US, e.g., shock), whereas the CS- remains unpaired with the US. An increase in the participant's fear response with the similarity between the GS and CS+ signifies fear generalization [29]. This study aims to explore the inhibitory effect of activating secure attachment on perceptual fear generalization. We hypothesized that activation of secure attachment could effectively block perceived fear generalization and that this inhibition would be significantly better than the effect of positive emotional priming. We also hypothesized that secure attachment priming exhibits stability in inhibiting fear generalization. Specifically, this inhibition remains unaffected by intolerance of uncertainty and attachment orientations.

## Methods

### Participants

Using G*Power 3.1, we estimated sample size, setting the Type I error probability at *α* = 0.05, test power at 1—*β* = 0.95, and effect size at *f* = 0.25, resulting in an estimated sample size of 28. For this study, we recruited 63 university students voluntarily through online platforms. All participants were right-handed, possessed normal or corrected-to-normal vision, had no history of psychiatric disorders, and had not previously participated in emotion-related experiments. Before the experiment, participants were briefed on the following: a) the application of mild electrical shocks to their right wrist during the experiment, with pre-adjusted intensity deemed harmless to the human body; b) their right to terminate the experiment at any discomfort; c) the strict confidentiality and research-exclusive use of all participant information and data. Participants who comprehended and consented to participate signed a written informed consent form before the experiment, and successful completions were rewarded with compensation. This study received approval from the Academic Ethics Committee of Guangdong University of Petrochemical Technology and was conducted in accordance with the Declaration of Helsinki.

Due to equipment issues and the failure to acquire fear learning, six participants were excluded from the study. Of these, two participants experienced data inaccuracies due to equipment malfunction, while four participants failed to acquire fear during the conditioning phase. The criterion for unsuccessful fear conditioning was a difference between the subjective expectancy of shock ratings and skin conductance responses (SCRs) for CS + and CS- in the final block of the conditioning phase equal to or less than 0. In the final analysis, data from 57 participants were included, consisting of 30 males and 27 females aged between 18 and 24 (*M* = 19.79, *SD* = 1.41).

### Materials

**Fig. 1:**
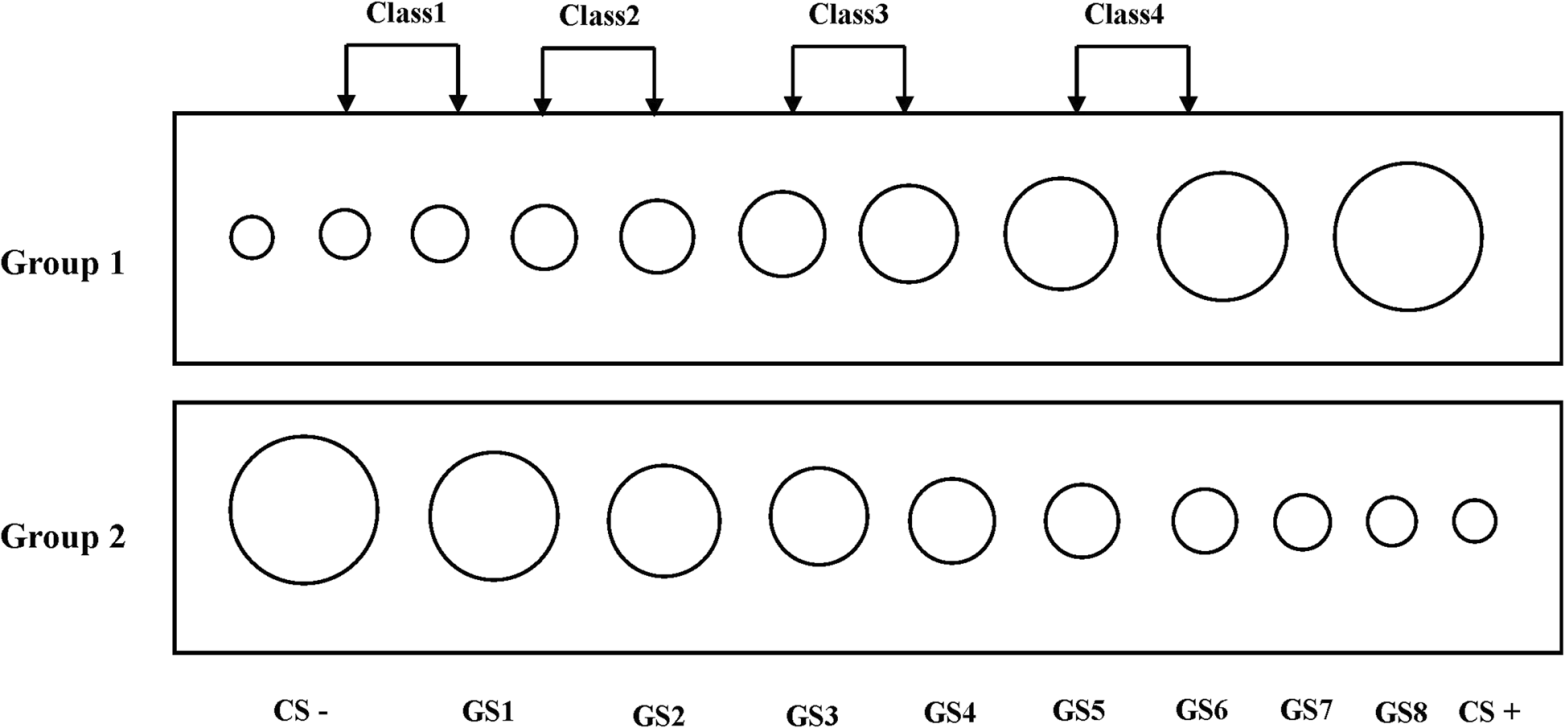
Conditioned stimuli and generalization stimuli materials. The conditioned stimulus materials were balanced across subjects

In line with the research conducted by Lissek et al. [28], a set of 10 circles served as stimuli for both conditioned (CS) and generalization (GS) purposes. These circles varied in size, with the smallest measuring 5.00 cm in diameter and the largest 11.75 cm. Assignment of the CS+ and CS- was based on the largest and smallest circles, respectively, ensuring balance across experimental groups. The remaining eight circles functioned as GS, each subsequent one expanding by 15% in diameter compared to the previous, equivalent to increments of 0.75 cm. To simplify the analysis, these GS were categorized into four levels (Class 1 through Class 4), with Class 1 consistently positioned closest to CS- and Class 4 closest to CS+ (Fig. 1). The unconditioned stimulus (US), potentially following CS+ , was administered using a constant-pressure stimulator (DS2A, Digitimer Ltd, UK), with each stimulus lasting 200 ms to elicit fear responses among participants. Stimulus intensity was determined based on participants' pre-experiment assessments, calibrated to meet the "extremely uncomfortable but tolerable" standards criterion, in line with prior research on fear conditioning [30].

### Measurement indicators

#### State-trait anxiety

Trait anxiety was assessed using the State-Trait Anxiety Inventory (STAI) developed and revised by Spielberger et al. [31]. This study utilized the Trait Anxiety subscale from the STAI (Form Y-II, T-AI), which consists of 11 negative and nine positive emotion items, primarily used to evaluate individuals' stable traits of anxiety and tension. The internal consistency coefficient (Cronbach's α) for the Trait Anxiety subscale in this study was 0.84.

#### Intolerance of uncertainty

Intolerance of uncertainty was measured using the short form of the Intolerance of Uncertainty Scale (IUS-SF) revised by Carleton et al. [32]. The IUS-SF consists of 12 items scored on a 5-point Likert scale, with higher total scores indicating lower tolerance for uncertainty. In this study, the Cronbach's α coefficient for this scale was 0.89.

#### Adult attachment

Adult attachment was measured using the Revised Adult Attachment Scale (ECR-R) developed by Fraley et al. [33]. The ECR-R consists of 36 items scored on a 7-point Likert scale (1 = "strongly disagree", 7 = "strongly agree"), assessing two dimensions: "attachment anxiety" and "attachment avoidance." Previous research has demonstrated the scale's good psychometric properties. This study's Cronbach's α coefficient for attachment anxiety and avoidance were 0.89 and 0.87, respectively.

#### Subjective expectancy of shock ratings

In the acquisition and generalization phases, participants were presented with stimuli accompanied by a prompt asking, "Likelihood of shock following?" They were directed to indicate their response using a keypress, with options ranging from 1 to 5. A higher numerical value denoted a greater subjective perception of the likelihood of a shock following the stimulus.

#### SCRs

Participants' SCRs were captured using a Biopac physiological recorder (Model MP160) operating at a sampling rate of 2000 samples per second. Ag/AgCl electrodes, each with a diameter of 4 mm, were affixed to the distal phalanges of the index and ring fingers of the participant's left hand and connected to the EDA module. Subsequently, the data underwent offline processing through AcqKnowledge 5.0 software. During analysis, the maximum value within a 5000 ms window post the presentation of CS (GS, GS+) stimuli was documented, while the average value within a 1000 ms window pre the presentation of CS (GS, GS+) stimuli served as the baseline. The disparity between these two values denoted the raw SCRs triggered by CS (GS, GS+). Raw skin conductance data underwent range correction, with SCRs below 0.02 μs being set to 0. A square root transformation was also applied to the corrected SCRs to mitigate data skewness.

### Experimental design and procedure

**Fig. 2:**
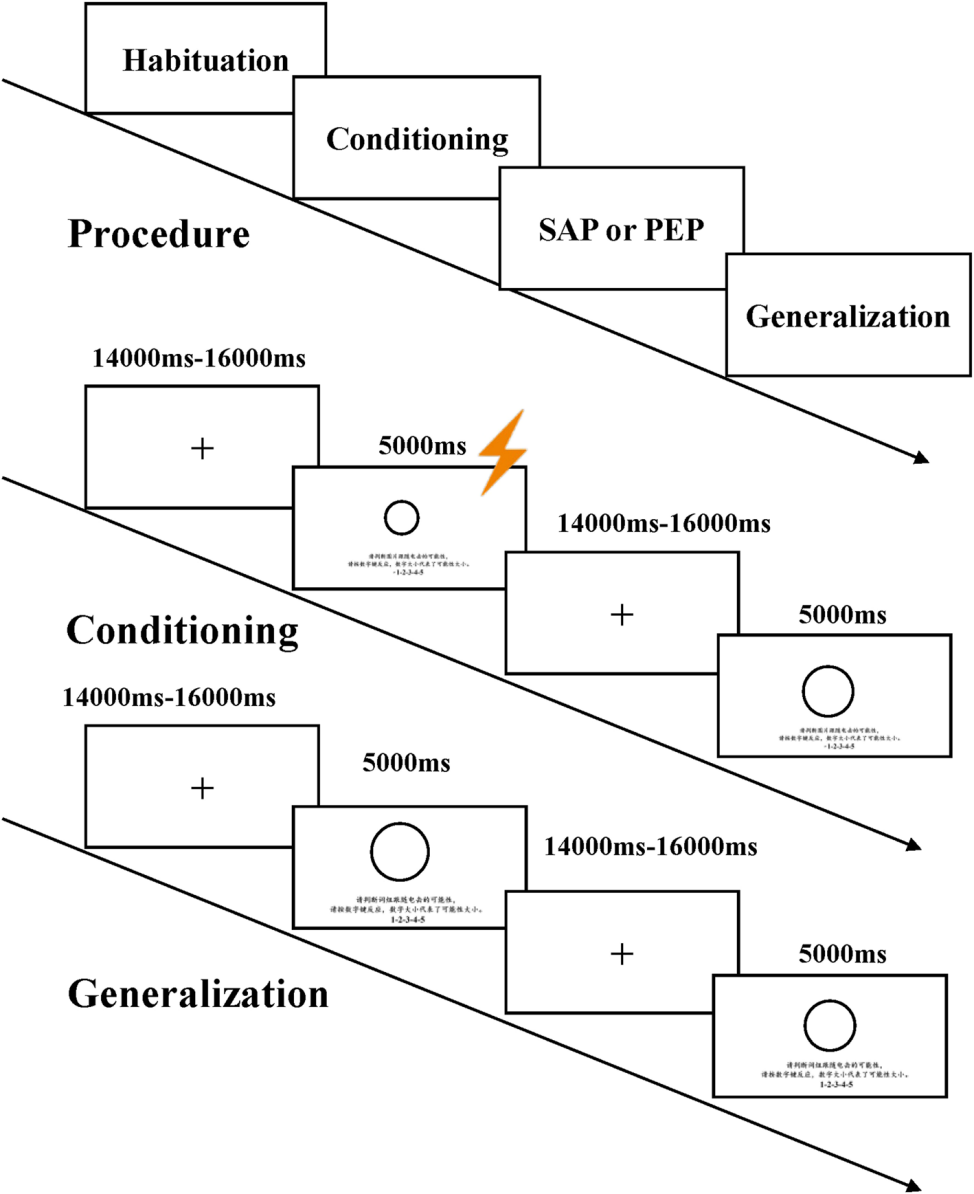
Overview of phases and experimental design. The procedure for the habituation phase was the same as for the conditioning phase, except that shocks did not accompany all the CS+ . SAP represents the secure attachment priming group, and PEP represents the positive emotion priming group

Following the fear generalization paradigm outlined by Lissek et al. [28], the experiment comprised four phases: pre-acquisition, acquisition, priming, and generalization, with the latter employing a block design (Fig. 2). These phases were conducted sequentially, with participants maintaining the connection of their left index and ring fingers to a physiological recorder, while their right hand was linked to a constant-pressure stimulator. Numeric keys on the keyboard were used for participant responses throughout the experiment.

Pre-acquisition phase: participants were exposed to large and small circles presented pseudo-randomly six times, with precautions taken to prevent consecutive presentations of the same stimulus more than twice. The main objective of this phase was to acquaint participants with the experimental protocols and gauge their initial responses to the stimuli. Notably, no shocks were administered subsequent to the presentation of the stimuli.

Acquisition phase: participants were exposed to both CS+ and CS- stimuli, which were presented pseudo-randomly twelve times each to prevent consecutive repetitions of the same CS more than twice. Subsequent to the presentation of CS+ , shock administration occurred with a 75% probability, while CS- was never followed by shock. Significant differences in the measurement indicators between CS+ and CS- indicate participants' successful induction of fear.

Priming phase: Following the study of Toumbelekis et al. [22], this phase comprised two conditions: secure attachment priming (experimental group) and positive emotion priming (control group). In the secure attachment priming group, participants were prompted to recall moments when a trusted individual provided support and assistance. They were then instructed to document this experience in writing and to immerse themselves in those feelings for a few minutes. Subsequently, participants rated the vividness, pleasure, and level of support and warmth experienced during the recalled scene using a 5-point scale. Conversely, the positive emotion priming group was directed to recall a pleasant scenic spot from a past trip while maintaining identical procedures to the experimental group.

Generalization phase: This phase consists of six blocks, each comprising 12 trials. In each block, the CS+ and CS- were each presented twice, while the GS occurred eight times, randomly distributed. One CS+ was followed by the US in each block to mitigate participant forgetfulness, whereas CS- and GS were not paired with shocks.

The experimental protocol was implemented using E-Prime 3.0. Each trial followed a specific sequence: initially, a red fixation cross, displayed at the screen's center for 14–16 seconds, aimed to focus participants' attention and allow SCRs to return to baseline levels. Subsequently, both the stimulus presentation and detection interface appeared simultaneously, prompting participants to assess the likelihood of a subsequent shock and respond via keyboard press. Following the participant's response, the stimulus interface remained visible for a maximum of 5000 ms. If a shock occurred after the stimulus presentation, it was presented 200 ms before the stimulus vanished, after which both the stimulus presentation and shock simultaneously disappeared. The experimental stimulus presentation process is depicted in Fig. 2.

### Data analysis

This study used a multifactor mixed design to conduct research, in which priming groups were used as between-subjects factors, and variables such as psychological test results, emotional indicators (vividness, pleasantness, and warmth), experimental materials, and generalization time points were used as within-subjects factors. Furthermore, this study used the moderation effect model to analyze the impact of individual differences on the fear generalization inhibition effect of different priming types [34]. In the moderation effect model, attachment orientations and intolerance of uncertainty were used as predictor variables, priming types as the moderating variables, and participants' average SCRs in the generalization phase across six blocks were used as the dependent variable [35]. The statistical analysis in this study was conducted using the *R* language and the *BruceR* package [36].

## Results

Independent sample *t*-tests were performed to compare anxiety levels among participants before the experiment to assess the effectiveness of randomization in participant allocation. During the pre-acquisition phase, a 2 × 2 repeated-measures ANOVA was conducted, with the group as the between-subjects variable and stimulus material (CS+ and CS-) as the within-subjects variable, to analyze SCRs.

The results indicate that there were no significant differences in trait anxiety (*t* (55) = -0.48, *p* > 0.05, Cohen’s *d* = -0.13) among participants from different groups. Regarding SCRs, there were no significant main effects for the group (*F* (1, 55) = 3.20, *p* > 0.05, partial *η*^*2*^ = 0.06) or stimulus material (*F* (1, 55) = 2.84, *p* > 0.05, partial *η*^*2*^ = 0.05), and the interaction between group and stimulus material was also not significant (*F* (1, 55) = 0.77, *p* > 0.05, partial *η*^*2*^ = 0.01). These results indicate no significant differences in fear responses to CS + and CS- between the two groups of participants before the formal experiment.

### Acquisition phase

To examine fear acquisition during the conditioning phase, a 2 × 2 repeated-measures ANOVA was conducted with the group as the between-subjects variable and stimulus material (CS+ and CS-) as the within-subjects variable, analyzing participants' subjective expectancy of shock ratings and SCRs.

In terms of expectancy of shock ratings, there was no significant main effect for the group (*F* (1, 55) = 3.08, *p* > 0.05, partial *η*^*2*^ = 0.05); while the main effect for stimulus material was significant (*F* (1, 55) = 139.63, *p* < 0.001, partial *η*^2^ = 0.72), the shock ratings were larger to the CS + versus CS-; and the interaction between group and stimulus material was not significant (*F* (1, 55) = 0.10, *p* > 0.05, partial *η*^*2*^ = 0.02). Regarding SCRs, there was no significant main effect for the group (*F* (1, 55) = 2.76, *p* > 0.05, partial *η*^*2*^ = 0.05); a significant main effect for stimulus material (*F* (1, 55) = 132.06, *p* < 0.001, partial *η*^*2*^ = 0.71), the SCR were larger to the CS + versus CS-; and the interaction between group and stimulus material was not significant (*F* (1, 55) = 2.13, *p* > 0.05, partial *η*^*2*^ = 0.04). These results indicate that both groups of participants successfully acquired fear during the conditioning phase.

### Priming phase

To investigate the effects of various priming methods among different groups, we conducted a 2 × 3 repeated-measures ANOVA, with the group as the between-subjects variable and priming indices (vividness, pleasure, and warmth) as the within-subjects variables.

The results indicate that there was no significant main effect for the group (*F* (1, 55) = 0.24, *p* > 0.05, partial *η*^*2*^ = 0.004). However, a significant main effect for emotional indices emerged (*F* (2, 110) = 4.15, *p* < 0.05, partial *η*^2^ = 0.07), along with a significant interaction between group and emotional indices (*F* (2, 110) = 5.00, *p* < 0.01, partial *η*^2^ = 0.08). Further analysis revealed no significant differences between the experimental and control groups in terms of the vividness of scene recall (*t* (55) = 0.60, *p* > 0.05, Cohen’s *d* = 0.18) and priming of positive emotion (*t* (55) = 0.21, *p* > 0.05, Cohen’s *d* = 0.06). However, a significant difference in warmth was observed (*t* (55) = -2.21, *p* < 0.05, Cohen’s *d* = -0.61), with the secure attachment priming group scoring significantly higher than the positive emotion priming group. These findings suggest that the secure attachment priming effect in the experimental group was significant compared to the control group [16, 17].

### Generalization phase

#### Degree of generalization

To evaluate the degree of fear generalization across various groups, we conducted a 2 × 6 repeated-measures ANOVA. The between-subjects factor was the group, while the within-subjects factor comprised the generalization stimuli (CS-, Class 1, Class 2, Class 3, Class 4, CS+). The primary objective of this analysis was to compare participants' expectancy of shock ratings and skin conductance responses (SCRs) to different generalization stimuli during the generalization phase.

##### Subjective expectancy of shock ratings

**Fig. 3:**
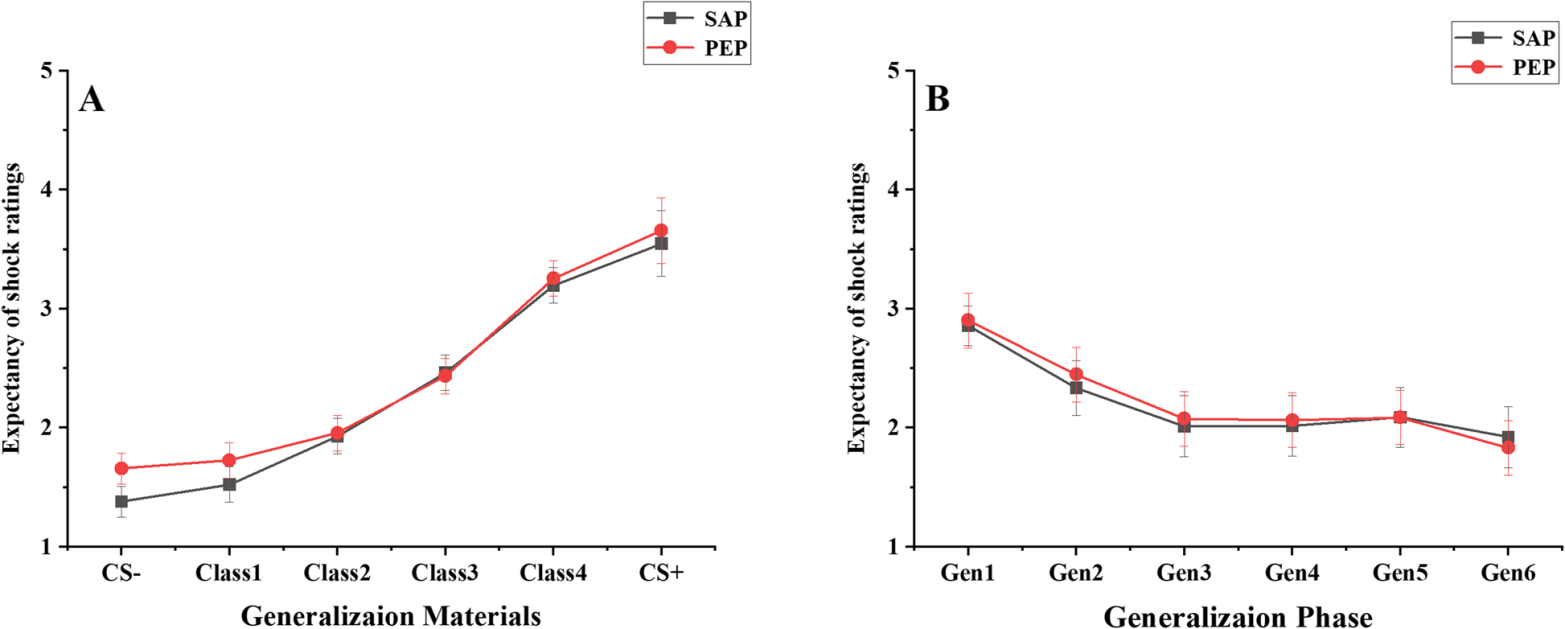
Inhibitory effects of different priming types on fear generalization (subjective expectancy of shock rating). Class 1- Class 4 represents different levels of generalized materials, with Class 1 consistently positioned closest to CS- and Class 4 closest to CS+ . Gen1-Gen6 represents the means of Class 1- Class 4 within Block1-Block6, respectively. SAP represents the secure attachment priming group, and PEP represents the positive emotion priming group

No significant main effect was found for the group (*F* (1, 55) = 0.23, *p* > 0.05, partial *η*^*2*^ = 0.01). However, there was a significant main effect for stimulus material (*F* (5, 275) = 106.78, *p* < 0.001, partial *η*^2^ = 0.66). Multiple comparison results show significant differences between all pairs of experimental materials (*t* (55) ≥ 3.6, *p* < 0.05, Cohen’s *d* ≥ 0.30). The interaction between the group and stimulus material was not significant (*F* (5, 275) = 0.20, *p* > 0.05, partial *η*^*2*^ = 0.01), suggesting no significant differences in the expectancy of shock ratings for various generalization materials between the two participant groups (see Table 1 and Fig. 3A).

**Table 1:** The descriptive statistical results of the generalization degree

Indicator	Group	CS-	CS+	Class 1	Class 2	Class 3	Class4
Shock ratings	SAP	1.32 (0.56)	3.69 (1.14)	1.65 (0.66)	1.90 (1.00)	2.15 (1.09)	3.08 (1.07)
	PEP	1.52 (0.80)	3.83 (0.68)	1.67 (0.690)	1.92 (0.69)	2.20 (0.83)	3.10 (0.78)
SCRs	SAP	0.41 (0.29)	0.61 (0.39)	0.37 (0.22)	0.39 (0.26)	0.39 (0.29)	0.48 (0.34)
	PEP	0.54 (0.37)	0.95 (0.52)	0.55 (0.34)	0.55 (0.35)	0.61 (0.36)	0.81 (0.43)

The data in the table are presented as mean (standard deviation). Class 1- Class 4 represents different levels of generalized materials, with Class 1 consistently positioned closest to CS- and Class 4 closest to CS + . SAP represents the secure attachment priming group, and PEP represents the positive emotion priming group. *N* = 57

##### SCRs

The analysis revealed significant main effects for group (*F* (1, 55) = 7.41, *p* < 0.01, partial *η*^2^ = 0.12), stimulus material (*F* (5, 275) = 25.85, *p* < 0.001, partial *η*^2^ = 0.32), and an interaction between group and stimulus material (*F* (5, 275) = 3.15, *p* < 0.05, partial *η*^2^ = 0.05), indicating disparities between participant groups across various generalization materials. Further simple effect tests revealed significant differences in the grouping factors in Class 1 (*t* (55) = 2.32, *p* < 0.05, Cohen’s *d* = 0.67), Class 3 (*t* (55) = 2.55, *p* < 0.05, Cohen’s *d* = 0.85), Class 4 (*t* (55) = 3.10, *p* < 0.01, Cohen’s *d* = 1.21), and CS + (*t* (55) = 2.72, *p* < 0.01, Cohen’s *d* = 1.26), marginally significant differences in Class 2 (*t* (55) = 1.99, *p* = 0.052, Cohen’s *d* = 0.61), and no significant differences in CS- (*t* (55) = 1.53, *p* > 0.05, Cohen’s *d* = 0.52). These findings suggest that compared to the control group, participants in the experimental group exhibited lower SCRs to Class 1, Class 2, Class 3, Class 4, and CS+ .

**Fig. 4:**
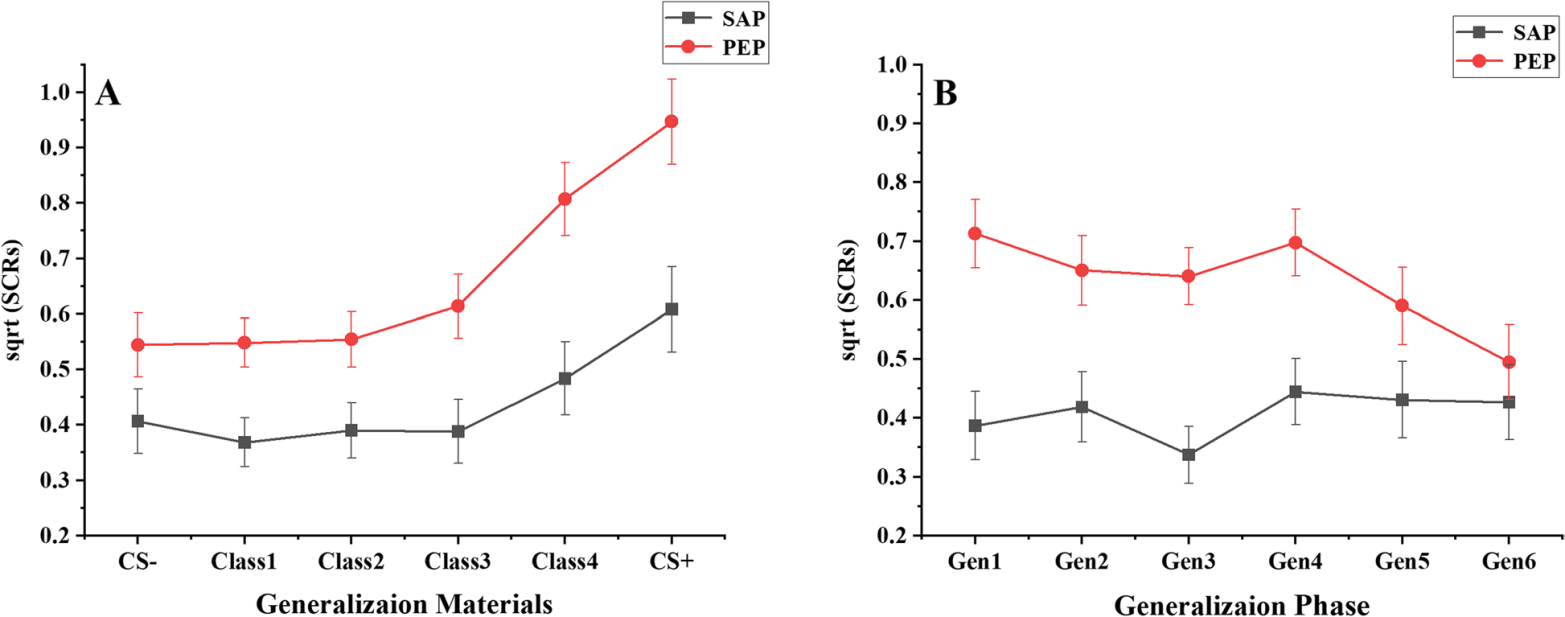
Inhibitory effects of different priming types on fear generalization (SCRs). Class 1- Class 4 represents different levels of generalized materials, with Class 1 consistently positioned closest to CS- and Class 4 closest to CS+ . Gen1-Gen6 represents the means of Class 1- Class 4 within Block1-Block6, respectively. SAP represents the secure attachment priming group, and PEP represents the positive emotion priming group

With CS- as the baseline, SCRs to Class 1, Class 2, Class 3, Class 4, and CS+ were individually assessed to elucidate the specific differences in fear generalization among different groups. The results indicated that participants in the experimental group exhibited no significant differences in SCRs to Class 1 (*t* (55) = -0.99, *p* > 0.05, Cohen’s *d* = -0.14), Class 2 (*t* (55) = -0.36, *p* > 0.05, Cohen’s *d* = -0.06), Class 3 (*t* (55) = -0.48, *p* > 0.05, Cohen’s *d* = -0.07), Class 4 (*t* (55) = 1.42, *p* > 0.05, Cohen’s *d* = 0.29), and CS + (*t* (55) = 2.67, *p* > 0.05, Cohen’s *d* = 0.76) compared to CS-, suggesting an absence of generalization phenomenon in SCRs for participants in the experimental group. Participants in the control group showed no significant differences in SCRs to Class 1 (*t* (55) = 0.11, *p* > 0.05, Cohen’s *d* = 0.02), Class 2 (*t* (55) = 0.24, *p* > 0.05, Cohen’s *d* = 0.04) and Class 3 (*t* (55) = 1.96, *p* > 0.05, Cohen’s *d* = 0.26) compared to CS-. However, they exhibited significantly higher fear activation responses to Class 4 (*t* (55) = 5.29, *p* < 0.001, Cohen’s *d* = 0.98) and CS + (*t* (55) = 5.82, *p* < 0.001, Cohen’s *d* = 1.51) compared to CS-, indicating fear generalization to Class 4 and CS + among participants in the control group (see Table 1 and Fig. 4A).

#### Generalization extinction

To investigate fear extinction status across different groups, a 2 × 6 repeated-measures ANOVA was conducted, with the group as the between-subjects variable and generalization time points (Gen1, Gen2, Gen3, Gen4, Gen5, Gen6) as the within-subjects variables. This analysis assessed participants' expectancy of shock ratings and SCRs at various generalization time points. Here, Gen1-Gen6 represents the means of Class 1- Class 4 within Block1-Block6, respectively.

##### Subjective expectancy of shock ratings

The analysis did not reveal any significant main effect for the group (*F* (1, 55) = 0.01, *p* > 0.05, partial *η*^*2*^ = 0.001). However, there was a notable main effect for the time process (*F* (5, 275) = 33.42, *p* < 0.001, partial *η*^2^ = 0.38). Multiple comparison results show significant differences between Gen1 and Gen2, Gen1 and Gen3, Gen1 and Gen4, Gen1 and Gen5, Gen1and Gen6, Gen 2 and Gen3, Gen2 and Gen4, Gen2 and Gen6,* t* (55) ≤ -3.78, *p* < 0.01, Cohen’s *d* ≤ -0.44. Furthermore, the interaction between the group and time process was not significant (*F* (5, 275) = 0.26, *p* > 0.05, partial *η*^*2*^ = 0.005), indicating no significant differences in the expectancy of shock ratings for generalization time points between the two groups of participants (see Table 2 and Fig. 3B).

**Table 2:** The descriptive statistical results of the generalization extinction

Indicator	Group	Gen1	Gen2	Gen3	Gen4	Gen5	Gen6
Shock ratings	SAP	2.89 (0.65)	2.28 (0.99)	2.01 (1.00)	2.01 (1.09)	2.07 (0.98)	1.94 (0.95)
	PEP	2.92 (0.72)	2.38 (0.82)	2.069 (0.87)	2.060 (0.68)	2.07 (0.81)	1.85 (0.59)
SCRs	SAP	0.39 (0.30)	0.42 (0.30)	0.34 (0.25)	0.45 (0.29)	0.43 (0.33)	0.43 (0.32)
	PEP	0.71 (0.43)	0.65 (0.46)	0.64 (0.41)	0.70 (0.39)	0.59 (0.39)	0.50 (0.41)

The data in the table are presented as mean (standard deviation). Gen1-Gen6 represents the means of Class 1- Class 4 within Block1-Block6, respectively. SAP represents the secure attachment priming group, and PEP represents the positive emotion priming group. *N* = 57

##### SCRs

A significant main effect was found for the group (*F* (1, 55) = 7.64, *p* < 0.01, partial *η*^2^ = 0.12), whereas the main effect for the time process was not significant (*F* (5, 275) = 1.87, *p* > 0.05, partial *η*^*2*^ = 0.03). However, a significant interaction between group and time process was observed (*F* (5,275) = 2.60, *p* < 0.05, partial *η*^2^ = 0.05). Further simple effects analysis revealed significant group differences for Gen1 (*t* (55) = 2.32, *p* < 0.05, Cohen’s *d* = 0.67), Gen2 (*t* (55) = 2.55, *p* < 0.05, Cohen’s *d* = 0.85), Gen3 (*t* (55) = 3.10, *p* < 0.01, Cohen’s *d* = 1.21), and Gen4 (*t* (55) = 2.72, *p* < 0.01, Cohen’s *d* = 1.26), with non-significant differences for Gen5 (*t* (55) = 1.53, *p* > 0.05, Cohen’s *d* = 0.50) and Gen6 (*t* (55) = 1.53, *p* > 0.05, Cohen’s *d* = 0.21). These findings indicate significant variations in SCRs between the two participant groups across generalization time points. Specifically, participants in the experimental group displayed lower SCRs at Gen1, Gen2, Gen3, and Gen4 than the control group (see Table 2 and Fig. 4B).

### Individual differences analyses

Differential generalization was observed only in SCRs between the experimental and control groups, so individual differences analyses were conducted solely on SCRs. The PROCESS() function (Model 1) in the *BruceR* package was utilized to examine the predictive influences of intolerance of uncertainty and attachment orientations across different priming groups. The study employed intolerance of uncertainty and different attachment orientations as predictor variables, respectively, with priming type as the moderating variable. The dependent variables were the average SCRs across Class 1 to Class 4 in the six blocks of the generalization phase [35].

**Fig. 5:**
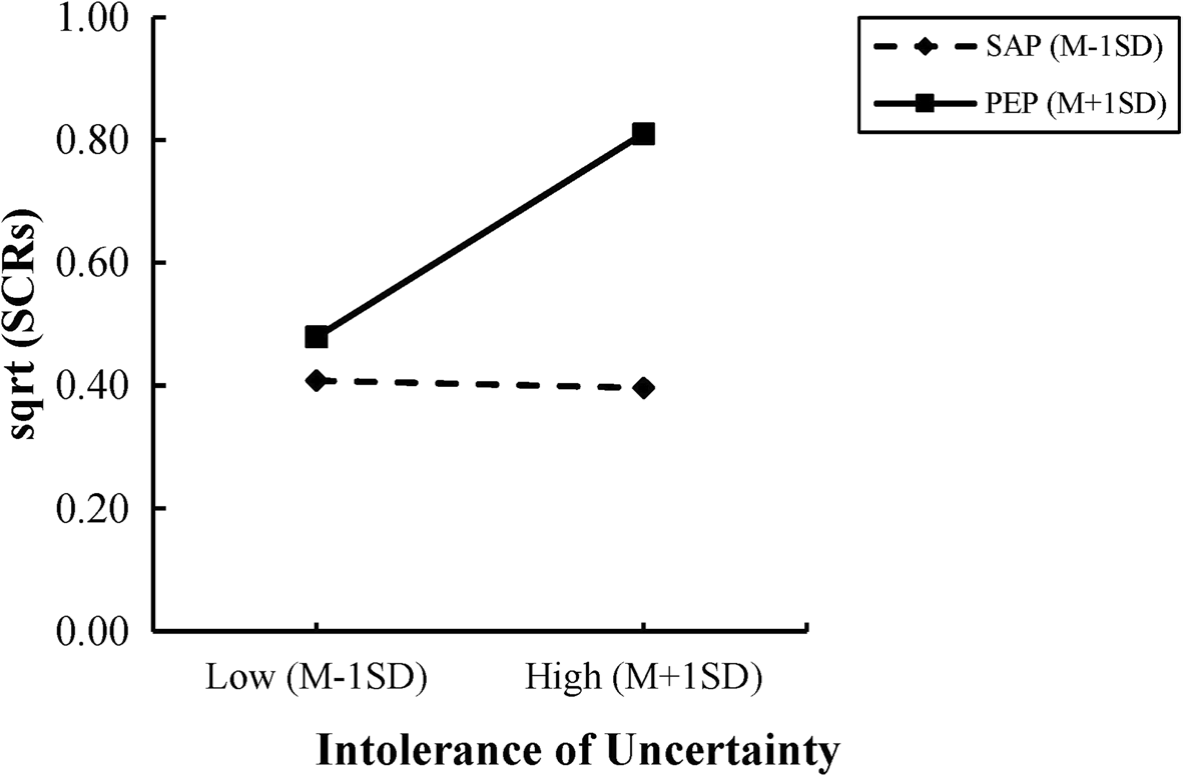
Individual differences analyses. SAP represents the secure attachment priming group, and PEP represents the positive emotion priming group

The analysis uncovered a significant impact of intolerance of uncertainty on the inhibition of priming type on generalization (*β* = 0.31, *p* < 0.05). Further examination through simple slope analyses indicated that the strength of the positive emotion priming effect decreased as intolerance of uncertainty increased (*t* (55) = 0.28, *p* < 0.01). In contrast, the effect of secure attachment priming remained unaffected by intolerance of uncertainty (*t* (55) = -0.03, *p* > 0.05) (Fig. 5). Additionally, the results suggest that attachment avoidance (*β* = 0.12, *p* > 0.05) and attachment anxiety (*β* = 0.07, *p* > 0.05) did not influence the inhibition of priming type on SCRs.

## Discussion

In recent years, investigating fear interventions from a social relationship perspective has become a research hotspot [37]. This study, grounded in the Integrative Model of Attachment-System Dynamics, explored the inhibitory effect of activated secure attachment representations on perceptual fear generalization. The results verified the inhibitory effect of secure attachment priming on perceptual fear generalization in terms of both the extent of generalization and generalization extinction. Specifically, secure attachment priming was found to reduce perceptual fear generalization physiologically, and this inhibitory effect was found to be independent of the effects of positive emotion priming. This finding holds significant implications for the specific guidance in developing clinical psychological interventions tailored to individuals who have experienced traumatic events.

### Secure attachment priming inhibits the generalization of fear

This study found that activating secure attachment representations effectively reduced the generalization of perceptual fear at the physiological level. In a discriminative fear conditioning paradigm, where CS+ signals impending danger or threat stimulus, and CS- represents a safety signal, individuals need to mobilize resources to cope with the threat represented by CS+ while inhibiting fear responses to CS-. Differential tests with CS- revealed that participants in the experimental group did not exhibit any generalization phenomenon in SCRs to the generalization materials, whereas participants in the control group generalized their responses to Class 4 and CS+ . Furthermore, over time, the generalization extinction in the experimental group was smoother compared to the control group, which showed a peak followed by a decline phenomenon. Specifically, the SCRs of participants in the experimental group were significantly lower than those in the control group across Gen1-Gen4, with no significant differences between the two groups in Gen5 and Gen6. These results are consistent with Toumbelekis et al.'s findings regarding the inhibitory effects of attachment priming on fear acquisition and extinction [22].

The attachment theory suggests that individuals, when confronted with physiological or psychological threats, view attachment figures as a "Safe Haven" for seeking protection and as a "Secure Base" for exploring the external world [38]. This study aimed to activate participants' secure attachment representations using imagery and found that it inhibited perceptual fear generalization. The findings demonstrate that imagery-based techniques can activate participants' secure base schemas, temporarily enhancing their sense of security [39]. When individuals face traumatic events like natural disasters or accidents, timely and effective interpersonal support or guiding them to imagine secure attachment scenes can prevent further fear generalization, thus averting the onset and progression of anxiety disorders.

### Secure attachment priming has inhibitory effects independent of positive emotions

Broaden-and-Build Theory posits that positive emotions broaden individuals' attention, enhancing receptivity and openness, thus increasing flexibility in problem-solving [40]. Research by Feng et al. has demonstrated that positive emotions facilitate the learning and generalization of safety signals, thereby inhibiting the generalization of conditioned fear [41]. This study further revealed that, compared to priming positive emotions, initiating secure attachment more effectively inhibits the extent of conditioned fear generalization and promotes its extinction. This finding underscores the independent influence of activating secure attachment representations on the priming effects of positive emotions.

Secure attachment priming temporarily alters individuals' access to positive and negative self-representations and representations of others, influencing their attachment security levels and subsequently impacting emotional processing. While secure attachment priming bears similarities to general positive emotion priming, its effects are not solely driven by the consistent emotions induced by priming materials [16]. Attachment priming paradigms engage behavioral, cognitive, and affective components of attachment representations [42]. The emergence of attachment priming effects is not solely attributed to the activation of positive or negative emotions in attachment schemas or representations but also involves the simultaneous activation of cognitive and affective components related to security in attachment schemas. Thus, when individuals experience physiological or psychological threat states, the effects of secure attachment priming surpass those of positive emotion priming [17].

### The inhibitory effect of secure attachment priming exhibits separation between subjective and physiological indices

The findings of this experiment reveal a distinct dissociation between subjective ratings and SCRs. Specifically, the activation of secure attachment demonstrates a significant inhibitory effect on perceptual fear generalization at the physiological arousal level, while the impact on subjective ratings is not significant. This outcome is consistent with the Dual-Process Model of fear learning [43], which proposes fear learning as the outcome of the interaction between two independent learning mechanisms. Subjective expectancy reflects explicit learning associations between conditioned stimuli (CS) and unconditioned stimuli (US), whereas SCRs represent implicit learning of CS-US associations [44]. Xu et al. identified gender differences in fear generalization, wherein the separation between physiological and subjective rating indices primarily manifests as differences in subjective fear ratings. Participants may not perceive fear at the physiological implicit level but tend to report higher fear expectancies subjectively [45]. Aligning with the results of Toumbelekis et al. [22], the dissociation of indices is also evident in this study, with no significant differences in subjective indices among participants but variations in SCRs as an indicator of implicit processing.

The inconsistency observed may originate from the secure attachment priming technique employed in this study, which was also utilized in the study conducted by TT et al. According to Mikulincer & Shaver [15], individuals' representations of attachment experiences are prelinguistic, constituting a specialized working model or mental schema formed in a procedural memory format. Secure attachment priming enhances the accessibility and permeability of these representations in individuals' long-term memory networks, making them highly active and easily retrievable [46]. The activation process of secure attachment representations is remarkably rapid, requiring minimal information processing resources and primarily guiding individuals' cognition, emotions, and behaviors in an unconscious manner, similar to responses exhibited by securely attached individuals [47]. In this study, the secure attachment priming technique reactivates participants' attachment schemas and influences their cognitive processing of fear information and emotional arousal in an unconscious, automatized manner.

### Individual differences do not affect the inhibition of secure attachment priming on fear generalization

This investigation incorporated intolerance of uncertainty and attachment orientations into the regression equation to examine the role of individual differences in the impact of various priming types on inhibiting fear generalization. The findings revealed that as intolerance of uncertainty increased, the impact of positive emotion priming decreased, whereas the influence of secure attachment priming remained unaltered by intolerance of uncertainty. Moreover, neither positive emotion priming nor secure attachment priming was affected by attachment orientations. These results indicate the robustness of secure attachment priming compared to positive emotion priming, suggesting that secure attachment priming can inhibit fear generalization regardless of individual anxiety levels. Furthermore, these results further prove that secure attachment priming has an effect independent of attachment orientations [17].

The activation of different attachment schemas through attachment contextual stimuli causes the attachment priming effect, which, in turn, triggers corresponding attachment strategies. There is controversy regarding whether an individual's attachment style influences the effect of secure attachment priming. Early studies suggested that the effect of secure attachment priming is not dependent on an individual's past attachment experiences [48]. Individuals with different attachment styles activate their inherent secure attachment representations in threatening situations to seek help and support, temporarily experiencing a sense of protection and safety. However, other studies have found that individuals with different attachment styles may adopt different attachment behavioral strategies and emotional regulation methods when the attachment system is activated, which can lead to the ineffectiveness of the secure attachment priming effect [49]. The results of this study support the former view, indicating that their trait attachment styles do not influence the secure attachment priming effects of individuals. This dissociation may stem from the differences in experimental paradigms between the two types of studies. Secure attachment priming in normal contexts may directly activate an individual's dominant attachment schema, such as secure attachment, hyperactivating, or deactivating cognitive emotion regulation strategies, thereby guiding subsequent psychological and behavioral processes.

On the other hand, secure attachment priming in threatening situations may not directly activate specific attachment schemas in individuals but instead prioritize the activation of individuals' innate secure base schema, which seeks attachment figure support and help [50]. Neurophysiological studies on secure attachment priming further explain this phenomenon, such as the presentation of secure attachment cues being able to reduce amygdala activity when facing threat [51] and decrease individuals' adrenergic stress response in the face of stress [52]. This result further reveals that secure attachment priming regulates individuals' response to environmental stimuli through bottom-up threat evaluation, thus reducing the need for higher-level regulatory functions.

## Significance and prospects

On the one hand, the results of this study further verified that secure attachment priming in threat situations is independent of positive emotional effect, and this effect is not affected by individual trait attachment style. On the other hand, the application of secure attachment priming to the inhibition of perceived fear generalization expands the application scope of secure attachment priming and has clinical guiding significance for the prevention and intervention of anxiety spectrum disorders. Specifically, for individuals predisposed to anxiety, offering timely and effective interpersonal support or triggering their internal attachment representations post-fear acquisition can hinder the spread of fear-related information, thereby forestalling the onset of anxiety disorders.

This study also presents certain limitations. For instance, the method of secure attachment priming exclusively employed the thought priming technique. Future research endeavors could diversify by exploring alternative methods, such as subliminal priming, to probe further the impact of secure attachment priming in curtailing conditioned fear generalization. The sample size in the individual differences analysis section of this study is small, and future research could explore the impact of individual differences on fear generalization through large-sample studies. This study focused on the inhibitory effects of secure attachment priming on perceptual fear generalization, subsequent investigations could delve into its influence on situational memory generalization. Furthermore, a more robust body of evidence and theoretical framework is warranted to elucidate the neural and endocrine mechanisms underlying the suppression of fear generalization by secure attachment priming.

## Conclusion

In summary, this study investigated the inhibitory impact of secure attachment priming on perceptual fear generalization. The findings revealed that: (1) activating participants' secure attachment representations through imagination effectively suppresses the generalization of perceptual fear; (2) this inhibitory effect of secure attachment priming is independent of the effect of positive emotion priming; (3) individual trait attachment styles do not modulate the inhibitory effect of secure attachment priming, and secure attachment priming can foster the adoption of secure base strategies among insecurely attached individuals; (4) secure attachment priming exerts an inhibitory influence solely on perceptual fear generalization at the physiological level.

### Supplementary Information

Supplementary Material 1.

## Data Availability

Data is provided within the supplementary information files.

## Abbreviations

SAP: Secure attachment priming group
PEP: Positive emotion priming group

## References

[1] LeDoux JE (2009). Emotion Circuits in the Brain FOCUS.

[2] Dymond S, Dunsmoor JE, Vervliet B, Roche B, Hermans D (2015). Fear generalization in humans: Systematic review and implications for anxiety disorder research. Behav Ther.

[3] Steimer T (2002). The biology of fear- and anxiety-related behaviors. Dialogues Clin Neurosci.

[4] Lissek S, Kaczkurkin AN, Rabin S, Geraci M, Pine DS, Grillon C (2014). Generalized anxiety disorder is associated with overgeneralization of classically conditioned fear. Biol Psychiat.

[5] Hornstein EA, Haltom KE, Shirole K, Eisenberger NI (2018). A unique safety signal: Social-support figures enhance rather than protect from fear extinction. Clin Psychol Sci.

[6] Dou H, Dai Y, Qiu Y, Lei Y (2022). Attachment voices promote safety learning in humans: A critical role for P2. Psychophysiology.

[7] Pan Y, Olsson A, Golkar A (2020). Social safety learning: Shared safety abolishes the recovery of learned threat. Behav Res Ther.

[8] Dai Y, Dou H, Lei Y (2023). The Presence of Others Inhibited Fear Generalization. Psychol Sci.

[9] Dou H, Zou L, Becker B, Lei Y (2021). Intranasal oxytocin decreases fear generalization in males, but does not modulate discrimination threshold. Psychopharmacology.

[10] Raby KL, Dozier M (2019). Attachment across the lifespan: Insights from adoptive families. Curr Opin Psychol.

[11] Boag EM, Carnelley KB (2012). Self-reported discrimination and discriminatory behaviour: The role of attachment security. Br J Soc Psychol.

[12] Shaver PR, Mikulincer M (2002). Attachment-related psychodynamics. Attach Hum Dev.

[13] Mikulincer M, Shaver PR (2003). The Attachment Behavioral System In Adulthood: Activation, Psychodynamics, And Interpersonal Processes. In Advanc Exp Soc Psychol..

[14] Mikulincer M, Shaver PR, editors. Attachment in adulthood: Structure, dynamics, and change. Guilford Publications; 2010.

[15] Mikulincer M, Shaver PR (2019). Attachment orientations and emotion regulation. Curr Opin Psychol.

[16] Mikulincer M, Gillath O, Halevy V, Avihou N, Avidan S, Eshkoli N (2001). Attachment theory and rections to others’ needs: Evidence that activiation of the sense of attachment security promotes empathic responses. J Pers Soc Psychol.

[17] Gillath O, Karantzas GC, Romano D, Karantzas KM (2022). Attachment security *priming: A meta-analysis*. Pers Soc Psychol Rev.

[18] Coan JA, Beckes L, Gonzalez MZ, Maresh EL, Brown CL, Hasselmo K (2017). Relationship status and perceived support in the social regulation of neural responses to threat. Social Cognitive and Affective Neuroscience.

[19] Morriss J, Bell T, Johnstone T, Van Reekum CM, Hill J (2019). Social domain based modulation of neural responses to threat: The different roles of romantic partners versus friends. Soc Neurosci.

[20] Toumbelekis M, Liddell BJ, Bryant RA (2018). Thinking of attachment figures blocks differential fear conditioning. Social Cognitive & Affective Neuroscience.

[21] Toumbelekis M, Liddell BJ, Bryant RA (2021). Secure attachment priming protects against relapse of fear in Young adults. Transl Psychiatry.

[22] Toumbelekis M, Liddell BJ, Bryant RA (2021). Secure attachment primes reduce fear consolidation. Depress Anxiety.

[23] Morriss J, Zuj DV, Mertens G (2021). The role of intolerance of uncertainty in classical threat conditioning: Recent developments and directions for future research. Int J Psychophysiol.

[24] Morriss J, Macdonald B, Van Reekum CM (2016). What is going on around here? Intolerance of uncertainty predicts threat generalization. PLoS ONE.

[25] Nelson BD, Weinberg A, Pawluk J, Gawlowska M, Proudfit GH (2015). An event-related potential investigation of fear generalization and intolerance of uncertainty. Behav Ther.

[26] Bauer EA, MacNamara A, Sandre A, Lonsdorf TB, Weinberg A, Morriss J, Van Reekum CM (2020). Intolerance of uncertainty and threat generalization: A replication and extension. Psychophysiology.

[27] Ahrens LM, Pauli P, Reif A, Mühlberger A, Langs G, Aalderink T, Wieser MJ (2016). Fear conditioning and stimulus generalization in patients with social anxiety disorder. J Anxiety Disord.

[28] Lissek S, Biggs AL, Rabin SJ, Cornwell BR, Alvarez RP, Pine DS, Grillon C (2008). Generalization of conditioned fear-potentiated startle in humans: Experimental validation and clinical relevance. Behav Res Ther.

[29] Lissek S, Rabin S, Heller RE, Lukenbaugh D, Geraci M, Pine DS, Grillon C (2010). Overgeneralization of conditioned fear as a pathogenic marker of panic disorder. Am J Psychiatry.

[30] Starita F, Kroes MC, Davachi L, Phelps EA, Dunsmoor JE (2019). Threat learning promotes generalization of episodic memory. J Exp Psychol Gen.

[31] Spielberger CD, Gonzalez-Reigosa F, Martinez-Urrutia A, Natalicio LF, Natalicio DS. The state-trait anxiety inventory. Revista Interamericana de Psicologia/Interamerican Journal of Psychology. 1971;5(3&4):145–58.

[32] Carleton RN, Norton MPJ, Asmundson GJ (2007). Fearing the unknown: A short version of the Intolerance of Uncertainty Scale. J Anxiety Disord.

[33] Fraley RC, Waller NG, Brennan KA (2000). An item response theory analysis of self-report measures of adult attachment. J Pers Soc Psychol.

[34] Hayes AF. Introduction to mediation, moderation, and conditional process analysis: A regression-based approach (3rd edition). Guilford publications; 2022.

[35] Dymond S, Cameron G, Zuj DV, Quigley M. Far from the threatening crowd: Generalisation of conditioned threat expectancy and fear in COVID-19 lockdown. Learn Behav. 2024. 10.3758/s13420-024-00625-4. 10.3758/s13420-024-00625-4 PMC11408548 38286957

[36] Bao H-W-S. bruceR: Broadly useful convenient and efficient R functions. R Package Version 0.8. https://cran.r-project.org/web/packages/bruceR/index.html.

[37] Hornstein EA, Craske MG, Fanselow MS, Eisenberger NI (2022). Reclassifying the unique inhibitory properties of social support figures: A roadmap for exploring prepared fear suppression. Biol Psychiat.

[38] Mikulincer M, Shaver PR (2017). Augmenting the Sense of Attachment Security in Group Contexts: The Effects of a Responsive Leader and a Cohesive Group. Int J Group Psychother.

[39] Carnelley KB, Bejinaru M-M, Otway L, Baldwin DS, Rowe AC (2018). Effects of repeated attachment security priming in outpatients with primary depressive disorders. J Affect Disord.

[40] Fredrickson BL (2001). The role of positive emotions in positive psychology: The broaden-and-build theory of positive emotions. Am Psychol.

[41] Feng B, Xu L, Zhang W, Chen T, Wang W, Zheng X (2017). The inhibitive effect of positive emotions on fear generalization. Acta Psychol Sin.

[42] Mikulincer M, Hirschberger G, Nachmias O, Gillath O (2001). The affective component of the secure base schema: affective priming with representations of attachment security. J Pers Soc Psychol.

[43] Wiens S, Öhman A (2002). Unawareness is more than a chance event: Comment on Lovibond and Shanks (2002). J Exp Psychol Anim Behav Process.

[44] Taschereau-Dumouchel V, Kawato M, Lau H (2020). Multivoxel pattern analysis reveals dissociations between subjective fear and its physiological correlates. Mol Psychiatry.

[45] Xu L, Xie X, Yan P, Li J, Zheng X (2018). Sex differences in fear generalization. Acta Psychol Sin.

[46] Canterberry M, Gillath O (2013). Neural evidence for a multifaceted model of attachment security. Int J Psychophysiol.

[47] Gillath O, Karantzas G (2019). Attachment security priming: A systematic review. Curr Opin Psychol.

[48] Beckes L, Simpson JA, Erickson A (2010). Of snakes and succor: Learning secure attachment associations with novel faces via negative stimulus pairings. Psychol Sci.

[49] Gokce A, Harma M (2018). Attachment anxiety benefits from security priming: Evidence from working memory performance. PLoS ONE.

[50] Gillath O, Sesko AK, Shaver PR, Chun DS (2010). Attachment, authenticity, and honesty: Dispositional and experimentally induced security can reduce self-and other-deception. J Pers Soc Psychol.

[51] Norman L, Lawrence N, Iles A, Benattayallah A, Karl A (2015). Attachment-security priming attenuates amygdala activation to social and linguistic threat. Social Cognitive and Affective Neuroscience.

[52] Bryant RA, Chan L (2015). Thinking of attachments reduces noradrenergic stress response. Psychoneuroendocrinology.

